# Identification of salivary metabolomic biomarkers for oral cancer screening

**DOI:** 10.1038/srep31520

**Published:** 2016-08-19

**Authors:** Shigeo Ishikawa, Masahiro Sugimoto, Kenichiro Kitabatake, Ayako Sugano, Marina Nakamura, Miku Kaneko, Sana Ota, Kana Hiwatari, Ayame Enomoto, Tomoyoshi Soga, Masaru Tomita, Mitsuyoshi Iino

**Affiliations:** 1 Department of Dentistry, Oral and Maxillofacial Plastic and Reconstructive Surgery, Faculty of Medicine, Yamagata University, Yamagata 990-9585, Japan; 2 Institute for Advanced Biosciences, Keio University, Tsuruoka, Yamagata 997-0052, Japan; 3 Department of Oral Science, Division of Orthodontics, Graduate School of Dentistry, Kanagawa Dental University, Yokosuka, Kanagawa 238-8580, Japan

## Abstract

The objective of this study was to explore salivary metabolite biomarkers by profiling both saliva and tumor tissue samples for oral cancer screening. Paired tumor and control tissues were obtained from oral cancer patients and whole unstimulated saliva samples were collected from patients and healthy controls. The comprehensive metabolomic analysis for profiling hydrophilic metabolites was conducted using capillary electrophoresis time-of-flight mass spectrometry. In total, 85 and 45 metabolites showed significant differences between tumor and matched control samples, and between salivary samples from oral cancer and controls, respectively (*P* < 0.05 correlated by false discovery rate); 17 metabolites showed consistent differences in both saliva and tissue-based comparisons. Of these, a combination of only two biomarkers yielded a high area under receiver operating characteristic curves (0.827; 95% confidence interval, 0.726–0.928, *P* < 0.0001) for discriminating oral cancers from controls. Various validation tests confirmed its high generalization ability. The demonstrated approach, integrating both saliva and tumor tissue metabolomics, helps eliminate pseudo-molecules that are coincidentally different between oral cancers and controls. These combined salivary metabolites could be the basis of a clinically feasible method of non-invasive oral cancer screening.

Oral cancer (OC) is defined as a malignant tumor of the oral cavity, and is the sixth most common cancer worldwide, with an annual incident of 400,000 new cases accounting for 4% of cancers in men and 2% of cancers in women^1^,^2^,^3^. More than 7000 cases of oral cancer are diagnosed each year in Japan alone. This number has been steadily increasing during recent decades, and the rate of increase is higher in Japan than in the United States and other Western countries. Conventional visual and tactile examination (CVTE) is still the most common way to detect OC because oral cancer occurs in areas that can be adequately visualized. However, accurate diagnosis of subtle symptoms of early OC and inflammatory lesions is still difficult^4^, leading to diagnosis of OC in advanced stages^5^,^6^,^7^ with low prognosis, despite advances in treatment, which have resulted in an overall 5-year survival rate of approximately 50%^1^,^6^,^7^,^8^,^9^.

Few clinically established biomarkers for detecting OC currently exist and open biopsy is presently the only assured criteria to confirm a diagnosis of cancer. Although open biopsy is effective to diagnose OC, this method provides definitive drawbacks, such as invasiveness. Thus, novel, adjunctive screening aids (devices or tests) are desperately needed. For example, commercially available handheld wide-field devices that emit light in variable wavelengths that can result in reflectance and/or autofluorescence of the oral mucosa have been heavily marketed to the dental communities as an inexpensive and rapid way of improving CVTE^10^. However, there are limited data supporting their ability to increase diagnostic accuracy or assist the decision-making process for clinically evident lesions^11^,^12^, and therefore more reliable, objective, and biologically relevant methods are necessary.

Molecular biomarkers are ideal for objective screening and diagnosis, enabling the early detection of OC^13^,^14^,^15^. Compared with biomarkers in blood^16^, salivary biomarkers have obvious advantages; sampling is non-invasive, convenient and safe, thus facilitating frequent screening for oral cancers. This fluid is also clinically important as it filters the blood, possibly reflecting systemic physiological conditions. However, conventional tumor markers, such as cancer squamous cell carcinoma (SCC) antigen and Cyfra 21-1 in both blood and saliva were not shown to be clinically accurate enough, especially in early stages^17^,^18^,^19^. For example, only 10.9% of early stage disease (stages I and II) and 46% of advanced stage disease (stages III and IV) were detected as positive by SCC-antigen level (SCC-antigen >2.0 ng/ml)^18^. Thus, more accurate biomarkers should be explored.

As salivary biomarkers for OC detection, mRNA, proteins, and microRNA have shown potential to be clinically important^20^,^21^,^22^,^23^. Salivary metabolomics is also emerging to diagnose or screen OC as well as leukoplakia and Sjogren’s syndrome^24^,^25^,^26^,^27^,^28^,^29^. The use of salivary metabolites is plausible because these molecules may be transferred into saliva by various cells, including OC, in the oral cavity and salivary glands. However, all salivary metabolomics studies have simply captured metabolomic phenotypes in saliva, and did not reveal the underlying biological mechanisms. As an example of using consensus changes in molecular expression in saliva and tissue, salivary microRNAs secreted from parotid gland tumors showed diagnostic potential for these tumors^30^. Such an approach could help exploring reliable salivary markers. Metabolic pathways in oral squamous cell carcinoma (OSCC) in cultured cells and tumor tissues have been quantified^31^,^32^. Concurrent analyses of saliva and tissue samples from identical subjects would help with identification of a link between them.

The aim of this study was to explore salivary metabolite biomarkers that could discriminate OC from healthy controls based on metabolomic analyses of saliva and OC tissue samples. Consistently observed aberrances in the metabolomic profiles between saliva and tissues were used to identify candidate biomarkers. A multiple logistic regression (MLR) model using salivary metabolite concentrations was developed and validated to access their discrimination ability for OC.

## Methods

### Study subjects

This study was conducted according to the Declaration of Helsinki principles. The study protocol was approved by the Ethics Committee of Yamagata University School of Medicine (2012-141). Written informed consent was obtained from each subject before participating in the study. Patients with oral cancer and healthy controls were recruited at the Department of Dentistry, Oral and Maxillofacial Plastic and Reconstructive Surgery of Yamagata University Hospital from 2012 to 2014. None had received any prior treatment such as chemotherapy or radiotherapy. All oral cancer patients provided both tumor tissues and saliva samples. No controls had a history of prior malignancy or autoimmune disorders.

### Collection of saliva and tissue samples

All samples were collected at 08:00am–12:00noon. Eating and drinking were not permitted for at least 1.5 hours prior to saliva collection. Each subject rinsed their mouth with water, and their saliva was collected in a 50 ml Falcon tube on ice. Approximately 400 μl unstimulated whole saliva was collected over 5–10 min. After collection, the saliva samples were immediately stored at −80 °C. All saliva samples were collected from the patients during hospitalization. At least 3 hours before collecting saliva, we confirmed subjects’ oral hygiene. When the dental-plaque and calculus deposit were remarkable, they were removed by using toothbrush without dentifrice and ultrasonic scaling.

Tissues were collected only from oral cancer patients. Parts of the resected specimens with radical excisions were collected as tumor and healthy tissues. The tumor tissue and healthy tissue consisted of the center of the resected malignant specimens and the farthest margin from the center of the resected malignant specimen, respectively. Both tissues were stored immediately at −80 °C.

### Metabolomic analysis of saliva and tissue samples

Frozen saliva was thawed at 4 °C for approximately 1.5 hours and subsequently dissolved using a Voltex mixer at room temperature and centrifuged through a 5-kDa cutoff filter (Millipore, Bedford, MA) at 9,100×g for at least 2.5 h at 4 °C; 45 μl of each sample was added to a 1.5 ml Eppendorf tube, with 2 mM of methionine sulfone, 2-[N-morpholino]-ethanesulfonic acid (MES), D-Camphol-10-sulfonic acid, sodium salt, 3-aminopyrrolidine, and trimesate.

For metabolite extraction, frozen tissue samples (approximately 50 mg) were plunged into methanol (625 μl) containing internal standards and 20 M each of methionine sulfone, D-camphor-10-sulfonic acid and 2-(n-morpholino)ethanesulfonic acid and homogenized at 1,500 rpm for 15 min using a Shake Master Neo (BMS, Tokyo, Japan) to inactivate the enzymes. Subsequently, 500 μl of chloroform and 200 μl of Milli-Q water were added to 500 μl of the homogenized solution, and the mixed solution was centrifuged at 4,600×*g* for 15 min at 4 °C. The upper aqueous layer (300 μl) was centrifugally filtered at 9,100×*g* for 3.5 hours at 4 °C through a 5-kDa cutoff filter (Millipore) to remove large molecules. The 150 μl filtrate was lyophilized and dissolved in 25 μl of Milli-Q water containing a reference compound (200 μM of 3-aminopyrrolidine and trimesate) prior to CE-time-of-flight (TOF)-MS analysis.

The instrumentation and measurement conditions used for CE-TOFMS were described elsewhere^33^,^34^ with slight modification. Detailed parameters and data processing are described online in the Supplementary Material and Methods.

Metabolite concentrations in saliva and tissues were evaluated by the Mann–Whitney test for saliva and the Wilcoxon matched-pairs signed-rank test for tissues. *P*-values for evaluating differences in metabolite concentrations between oral cancer and controls were corrected by false discovery rate (FDR) for considering multiple independent tests. For the other parameters, Mann–Whitney and Chi-square tests were used for quantitative and qualitative variables, respectively.

To access the discrimination ability of a combination of multiple salivary metabolites, a multiple logistic regression (MLR) model was developed. First, metabolites that showed adjusted *P* < 0.05 between tissue samples and matched control samples were selected. Second, metabolites that showed adjusted *P* < 0.05 in saliva samples from the cancer patients and healthy controls and also identical increasing or decreasing trends based on fold change of averaged concentrations were selected. Third, support vector machine-feature selection (SVM-FS) ranked the discrimination ability of each metabolite. Finally, stepwise feature selection (*P* = 0.05 for both forward and backward) was used among 10 top-ranked metabolites.

To access the generalization ability of the developed model, we conducted a *k*-fold cross-validation (CV; *k* = 5, 10, 15, and 20) 200 times with various random values. A resampling test—randomly selecting individuals to yield virtual cohorts including an identical number of subjects (*n* = 68), allowing redundant selection—was also conducted 200 times to eliminate optimistic results. For resampled data, the MLR models were evaluated. As another numerical validation, we randomly split data into training and validation datasets (each dataset included almost 50% of the data), and trained MLR models using the training dataset (i.e., determined the coefficients and intercept of the models), and subsequently validated the trained models using the validation datasets. These procedures were conducted 200 times with different random values.

The analyses were conducted using R (ver. 3.2.3)^35^, JMP (ver. 12.0.0.1, SAS Institute Inc., Cary NC), GraphPad Prism (ver. 5.0.2, GraphPad Inc., San Diego, CA), and MeV TM4 software (ver. 4.9.0)^36^.

## Results

### Subject characteristics

Table 1 shows the distribution of characteristics of subjects who provided saliva (*n* = 68). No significant differences were observed for age, sex, and periodontitis; however, the ratio of smokers in the OC group was significantly higher than the controls (*P* = 0.002). Further, 88% of the histological types of the cancers were OSCC. Among the OC patients, 18 subjects provided both saliva and matched tumor tissues; their characteristics are listed online in Supplementary Table S1.

### Metabolomic profile of oral cancer tissues

**Figure 1:**
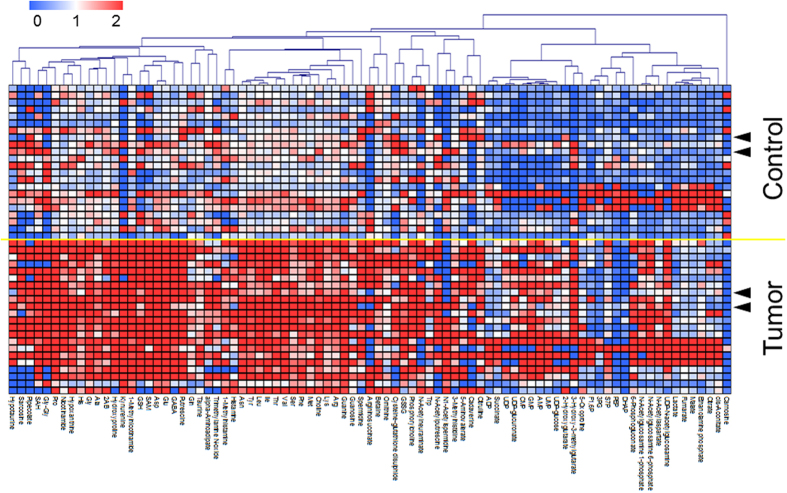
Metabolomic profile of matched tumor and control tissues. Each metabolite was normalized by dividing by the average of control samples. Samples colored in the red–white–blue scheme indicate relatively higher, average, and lower concentrations, respectively. Only metabolites showing a significant difference in adjusted *P* < 0.05 were used. These metabolites were clustered using Pearson correlation. Samples with triangles were collected from patients diagnosed with malignant melanoma; samples without triangles were collected from patients diagnosed with oral squamous cell carcinoma.

The heat map of metabolomic profiles of matched tumor and control tissues (Fig. 1) showed a clear distinction between the two groups. No histological type-specific difference was observed in these profiles. These data were also depicted in a metabolic pathway form (Supplementary Fig. S1). The concentration of lactate, an end product of glycolysis, was significantly elevated, whereas other intermediate metabolites, e.g. glyceraldehyde 3-phosphate (3PG) and phosphoenolpyruvate (PEP) were significantly decreased in the OC group. All metabolites in the urea cycle (e.g. arginine and ornithine) and one carbon cycle (e.g. *S*-Adenosylmethionine and *S*-adenosylhomocysteine), except homocysteine, were significantly increased in the OC group.

### Metabolomic profile of salivary samples

**Figure 2:**
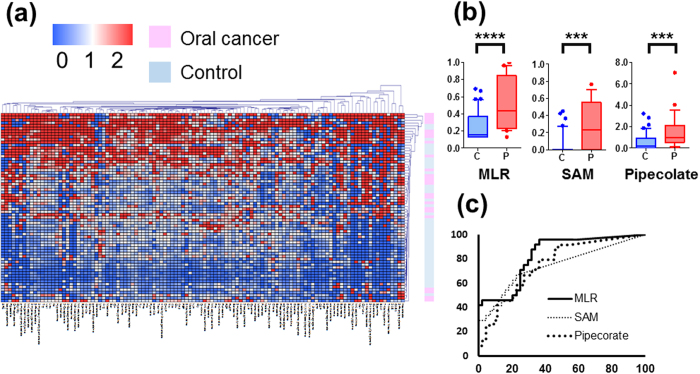
Metabolomic profiles in saliva. (**a**) A heat map shows metabolomic profiles in saliva samples. Both metabolites and samples were clustered using elucidation distance. Light pink and blue indicate oral cancer and controls, respectively. Samples colored in the red–white–blue scheme indicate relatively higher, average, and lower concentrations, respectively. Metabolites detected in ≥60% of either oral cancer or controls were used. (**b**) Probability of oral cancer using an MLR model and concentrations of *S*-adenosylmethionine (SAM) and pipecolate. ****P < 0.0001, ***P < 0.001. (**c**) ROC curves of data in (**b**) to differentiate oral cancer patients from healthy controls.

In the heat map of salivary metabolomic profiles, the OC profiles included higher concentrations of 98 metabolites (87.5% among 112 metabolites; Fig. 2a). Eighty-five tumor metabolites and 43 saliva metabolites showed significantly different concentrations between OC and controls, (*P* < 0.05 adjusted by FDR); in total, 17 metabolites showed significantly higher average concentrations consistently in both saliva and tissues (Supplementary Tables S2, S3, S4, Fig. S2). Of these metabolites, a combination of *S*-adenosylmethionine (SAM) and pipecolate using MLR yielded 0.827 (95% confidential interval [CI], 0.726–0.928, *P* < 0.0001) (Fig. 2b,c and Supplementary Table S5). The median of areas under receiver operating characteristic curve (AUC) of 200 *k*-fold CV iterations was robust; 0.805 (95% CI, 0.801–0.805), 0.804 (95% CI, 0.802–0.804), 0.804 (95% CI, 0.803–0.805), and 0.804 (95% CI, 0.803–0.804) for *k* = 5, 10, 15, and 20, respectively (Supplementary Fig. S3a). The resampled analyses yielded 0.841 (95% CI, 0.831–0.845) and 0.812 (95% CI, 0.798–0.814) using whole data and CV, respectively (Supplementary Fig. S3b). The data split analysis yielded 0.826 (95% CI, 0.821–0.837) and 0.826 (95% CI, 0.804–0.824) using training and validation datasets, respectively. The AUC values between training and validation did not showed significant differences (*P* = 0.138, Mann–Whitney test) (Supplementary Fig. S3c). Salivary metabolite markers did not significantly differ in comparison of early (stages I and II) and advanced (stages III and IV) stages of cancer (Supplementary Table S4).

## Discussion

In this study, we analyzed the metabolites from specimens of OC and identified salivary metabolites showing similar trends in saliva and tissue samples. Despite many salivary biomarker discoveries, most previous studies did not address the simple question: where do the salivary metabolites come from? Previous salivary metabolomic studies only captured disease-specific patterns and named signatures^24^,^29^. The approach shown here helps eliminate pseudomolecules that are coincidentally different between oral cancer and controls.

Metabolism in oral-cancer tissue was obviously different from healthy controls (Fig. 1). Many metabolites in primary pathways were different (Supplementary Fig. S1). The reduction of intermediate metabolites in glycolysis, such as 3PG (fold change [FC] = 0.76, adjusted *P* = 0.028) and PEP (FC. = 0.53, adjusted *P* = 0.029) while accumulation of lactate, an end product of glycolysis (FC = 1.64, adjusted *P* = 0.00089) indicated a Warburg effect (Supplementary Table S2). Glutamine increased (FC = 1.38, adjusted *P* = 0.028) while its elevation was less than glutamic acids (FC = 4.24, adjusted *P* = 0.000013), which was confirmed to be significantly elevated in the latter half of the TCA cycle, and also included succinate, fumarate, and malate, e.g. downstream of glutaminolysis via α-ketoglutarate (Supplementary Table S2). The activation of glycolysis and glutaminolysis metabolism was consistent with other reports^31^. The majority of the other metabolites in tumor tissues showed higher concentrations, especially kynurenine, a metabolite synthesized from tryptophan, which showed the highest fold change (FC = 38.1, adjusted *P* = 0.00027; Supplementary Table S2), indicating increased reactive oxygen species stress in tumor tissues^37^.

In our study, 17 metabolites showed consistently significant differences in both saliva and tissue samples (Supplementary Table S4). Most of these metabolites were consistent with previously reported salivary biomarkers. For example, choline and pipecolate detected OSCC even in early stages (I and II)^28^. SAM and methionine were intermediate metabolites in one-carbon metabolites starting from choline. SAM plays a pivotal role as a methyl donor^38^, being formed from methionine and converted into SAH after donating its methyl group^39^. Blockage of polyamine synthesis can result in a significant reduction of cancer proliferation rates in various cancers and therefore, alpha-difluoromethylornithine (DFMO) has been clinically evaluated for inhibiting the rate limiting enzyme of polyamine synthesis, ornithine decarboxylase (ODC) [EC 4.1.1.17]^40^. SAM also tightly regulates polyamine synthesis and serves as the sole donor of an aminopropyl group that conjugates with putrescine to form the polyamine spermidine, and then spermine^38^. Polyamines have been reported as biomarkers for various cancers^25^,^41^,^42^,^43^. In our study, spermidine was consistently elevated in both saliva and tissues. This metabolite is also a candidate biomarker for metabolism in these pathways.

Generally, cancer screening biomarkers should detect the malignancy at an early stage. Use of biomarkers to detect advanced-stage OC from biofluid is unnecessary, because advanced oral cancers are easily detected through CVTE. Furthermore, early detection of OC may improve patient survival outcomes, as the overall survival rate of patients with advanced cancer is generally poor. Several biomarkers such as SCC antigen for OC already exist. The presence of elevated SCC-Ag levels is reportedly associated with later-stage malignancy^17^,^18^,^19^,^44^,^45^,^46^. In our data, salivary markers did not show a stage-specific difference (Supplementary Tables S4). The metabolomic profiles in tumor tissue also offered no clear difference among patients, even in various stages and histological types, including OSCC, oral malignant melanoma (MM), and the status of periodontal diseases (Fig. 1, Supplementary Table S1). Stage-independent elevation of salivary metabolite markers, including choline and pipecolate, were also reported in another study^28^. These findings indicate that salivary metabolite biomarkers are clinically useful for screening or detecting OC patients, but not for diagnosis of disease stage.

There are several limitations of this study to be acknowledged. For instance, we selected salivary biomarkers based on the hypothesis that salivary and tumor metabolites show a positive correlation; however, tracing analyses that use isotope-labeled molecules more accurately show relationships between salivary and tumor metabolites.

In this study, we followed an established protocol for collecting unstimulated whole saliva, which has been used for various *omics* studies^47^,^48^,^49^. In this protocol, all subjects were asked to rinse their mouth with water before collection. This procedure might reduce oral bacteria, but at the same time, could cause saliva dilution, which would reduce the discrimination sensitivities for identified markers. Dental plaque is also a unique metabolic site and only rinsing is not enough to eliminate the effect of plaque on salivary metabolomic profiles^50^. Further study is needed to understand the relationship between plaque and salivary metabolites, by quantifying plaque levels^50^.

The cancer metabolism observed in OC tissue was not specific and is commonly observed in the other cancers, such as colon and stomach cancers^51^. Our previous salivary metabolomics studies also showed a large overlap of aberrant metabolites in oral, breast, and pancreatic cancers^25^, and the effects of smoking on salivary metabolites^34^. For example, salivary metabolites in choline and polyamine pathways (Supplementary Tables S2) had also been suggested as potential biomarkers for oral and breast cancers, respectively^28^,^43^,^52^. Pipecolate is significantly elevation in OC only compared with breast cancer, and with pancreatic cancer (in which it is decreased)^25^. The current study and the previous study showed a discrepancy^25^; SAM was detected only in the current data, possibly because of differences in storage duration of collected saliva samples. Storage conditions, such as duration and temperature, should be investigated for their effects on stability of identified markers^13^. Although SAM was identified as a possible marker in this study, this compound is also used for oral-administered agents for mental health, such as depression^53^. No subjects in this study used this agents; however, the effect its use should be evaluated. A validation study that includes a large group of subjects with various cancers and diseases, such as oral lichen planus and oral leukoplakia^29^, is necessary to evaluate the specificity of the identified biomarkers. Although CV showed consistent AUC values of the MLR model, these values in a resampled cohort fluctuated (Supplementary Fig. S3a and Fig. S3b), indicating a larger cohort validation is necessary to rigorously evaluate the accuracy of these biomarkers. Especially, evaluation using independently collected data is required to validate the discrimination ability of the model. The data-split analysis showed no significant difference in AUC values between training and validation datasets (Supplementary Fig. S3c), which suggests that the trained model is not over-fitted, and would discriminate oral cancer from controls with similar accuracy.

In summary, metabolomic profiles from tumor tissues were significantly different from healthy tissues. However, they were similar to salivary metabolites, which were significantly different between OC and control groups. Our research will be useful to understand the origin of salivary metabolites in OC patients. Despite the ability of the identified biomarkers to discriminate OC from controls, no significant difference in disease stages and histological types were identified, which prevents the use of these markers as screening tools to evaluate all OC.

## Additional Information

**How to cite this article**: Ishikawa, S. *et al*. Identification of salivary metabolomic biomarkers for oral cancer screening. *Sci. Rep*. **6**, 31520; doi: 10.1038/srep31520 (2016).

## Supplementary Material

Supplementary Information

## Figures and Tables

**Table 1:** Characteristics of subjects.

Parameter		Oral cancer	Control	*P*-value	
n	24	44	—		
Age	Median	72 (23–94)	68 (21–90)	0.542	
Sex	Male	14 (58.0)	16 (36.4)	0.123	
	Female	10 (42.0)	28 (63.6)		
Smoking habit	Yes	14 (58.3)	9 (20.5)	0.002	*
Periodontitis	Yes	16 (66.7)	29 (65.9)	0.584	
Stage	I	5 (21.0)		—	
	II	6 (25.0)			
	III	8 (33.0)			
	IV	5 (21.0)			
Histological type	Squamous cell carcinoma	21 (88.0)		—	
	Malignant melanoma	2 (8.0)			
	Unknown	1 (4.0)			

Note: Parentheses show ranges for age, and percentages of each group for the other parameters.
