# Supplementary material for: Identification of salivary metabolomic biomarkers for oral cancer screening
Source: Sci Rep. 2016 Aug 19;6:31520. doi: 10.1038/srep31520 (PMC4990923; doi:10.1038/srep31520)
Supplement: Supplementary Information [file srep31520-s1.doc]

Supplementary Information

Identification of salivary metabolomic biomarkers for oral cancer screening

Shigeo Ishikawa^1^, Masahiro Sugimoto^2,3*^, Kenichiro Kitabatake^1^, Ayako Sugano^1^, Marina Nakamura^1^, Miku Kaneko^2^, Sana Ota^2^, Kana Hiwatari^2^, Ayame Enomoto^2^, Tomoyoshi Soga^2^, Tomita Masaru^2^ and Mitsuyoshi Iino^1^

^1^Department of Dentistry, Oral and Maxillofacial Plastic and Reconstructive Surgery, Faculty of Medicine, Yamagata University, Yamagata 990-9585, Japan

^2^Institute for Advanced Biosciences, Keio University, Tsuruoka, Yamagata 997-0052, Japan

^3^Department of Oral Science, Division of Orthodontics, Graduate School of Dentistry, Kanagawa Dental University, Yokosuka, Kanagawa 238-8580, Japan

**To whom correspondence should be addressed:** Masahiro Sugimoto, Ph.D

Institute for Advanced Biosciences, Keio University 246-2 Mizukami, Kakuganji, Tsuruoka, Yamagata 997-0052, Japan

Tel: +81-235-29-0528

Fax: +81-235-29-0574

E-mail: [msugi@sfc.keio.ac.jp](mailto:msugi@sfc.keio.ac.jp)

Material and Methods

## Measurement of instrument parameters

Cation analysis was performed using an Agilent CE capillary electrophoresis system (G1600AX), an Agilent G1969A LC/MSD TOF system, an Agilent 1100 series isocratic HPLC pump, a G3251A Agilent CEMS adapter kit, and a G1607A Agilent CE-ESI-MS sprayer kit (Agilent Technologies, Waldbronn, Germany). Anion analysis was performed using an Agilent CE capillary electrophoresis system (G1600AX), an Agilent G1969A LC/MSD TOF system, an Agilent 1100 series isocratic HPLC pump, a G3251A Agilent CE-MS adapter kit, and a G1607A Agilent CE-electrospray ionization (ESI) source-MS sprayer kit (Agilent Technologies). For the cation and anion analyses, the CE-MS adapter kit included a capillary cassette that facilitates thermostatic control of the capillary. The CE-ESI-MS sprayer kit simplifies coupling of the CE system with the MS system and is equipped with an electrospray source. For system control and data acquisition, Agilent ChemStation software for CE (A10.02) and Agilent MassHunter software for TOF-MS (B.02.00) were used. The original Agilent SST316Ti stainless steel ESI needle was replaced with a passivated SST316Ti stainless steel and platinum needle (passivated with 1% formic acid and a 20% aqueous solution of isopropanol at 80°C for 30 min) for anion analysis.

For cationic metabolite analysis using CE-TOFMS, sample separation was performed in fused silica capillaries (50 µm i.d. × 100 cm total length) filled with 1 mol/l formic acid as the running electrolyte. The capillary was flushed before each sample injection with formic acid (1 mol/l) for 20 min before the first use and for 4 min for tissue sample analyses, 5 min at ammonium formate, 5 min at Milli-Q and 5 min at run buffer for saliva sample analyses. Sample solutions (approximately 3 nl) were injected at 50 mbar for 5 s and a voltage of 30 kV was applied. The capillary temperature was maintained at 20°C and the temperature of the sample tray was kept below 5°C. The sheath liquid, composed of methanol/water (50% v/v) and 0.1 µmol/l hexakis(2,2-difluoroethoxy) phosphazene (Hexakis), was delivered at 10 µl/min. ESI-TOF-MS was conducted in the positive ion mode. The capillary voltage was set at 4 kV and the flow rate of nitrogen gas (heater temperature = 300°C) was set at 7 psig. In TOF-MS, the fragmentor, skimmer and OCT RF voltages were 75, 50 and 125 V, respectively. Automatic recalibration of each acquired spectrum was performed using reference standards ([13C isotopic ion of protonated methanol dimer (2MeOH + H)]^+^, *m/z* 66.0631) and ([protonated Hexakis (M + H)]^+^, *m/z* 622.0290). Mass spectra were acquired at a rate of 1.5 cycles/s over a m/z range of 50–1,000.

For anionic metabolite analysis using CE-TOFMS, a commercially available Cosmo(+) capillary (50 µm × 105 cm, Nacalai Tesque, Kyoto, Japan), chemically coated with a cationic polymer, was used for separation. Ammonium acetate solution (50 mmol/l; pH 8.5) was used as the electrolyte for separation. Before the first use, the new capillary was flushed successively with the running electrolyte (pH 8.5), 50 mmol/l acetic acid (pH 3.4), and then the electrolyte again for 10 min each. Before each injection, the capillary was equilibrated for 2 min by flushing with 50 mmol/l acetic acid (pH 3.4), and then with the running electrolyte for 5 min. A sample solution (approximately 30 nl) was injected at 50 mbar for 30 s, and a voltage of −30 kV was applied. The capillary temperature was maintained at 20°C and the sample tray was cooled below 5°C. An Agilent 1100 series pump equipped with a 1:100 splitter was used to deliver 10 μl/min of 5 mM ammonium acetate in 50% (v/v) methanol/water, containing 0.1 μM Hexakis, to the CE interface. Here, it was used as a sheath liquid surrounding the CE capillary to provide a stable electrical connection between the tip of the capillary and the grounded electrospray needle. ESI-TOF-MS was conducted in the negative ionization mode at a capillary voltage of 3.5 kV. For TOF-MS, the fragmentor, skimmer and OCT RF voltages were set at 100, 50 and 200 V, respectively. The flow rate of the drying nitrogen gas (heater temperature = 300°C) was maintained at 7 psig. Automatic recalibration of each acquired spectrum was performed using reference standards ([13C isotopic ion of deprotonated acetic acid dimer (2 CH3COOH–H)]^─^, *m/z* 120.03841), and ([Hexakis deprotonated acetic acid (M + CH3COOH–H)]^─^, *m/z* 680.03554). Exact mass data were acquired at a rate of 1.5 spectra/s over a *m/z* range of 50–1,000.

## Processing of metabolomics data

CE-TOFMS raw data were analyzed with our proprietary software, MasterHands. The peaks were identified by matching corresponding *m/z* values and normalized migration times of standard compounds. Metabolite concentration was calculated based on the combination of a mixture of standard compounds with mixed internal standards. Metabolite concentrations in tissue samples were divided by the weight of each tissue sample.

**Figure S1.** Metabolic pathway visualization from metabolomic profiles in tissue samples. Filled and open dots indicate the tumor and control tissues, respectively. Asterisks show adjusted *P*-values: **P* < 0.05, ***P* < 0.01, and ****P* < 0.001.

**Figure S2.** Heat map of metabolomic profiles of salivary samples. Orange: metabolites showing significant differences (adjusted *P* < 0.05). Red: higher concentration; white: average concentration; blue: lower concentration. Only metabolites were clustered using Pearson correlation.

**Figure S3.** Generalization test. **A)** AUC values from 200 times of cross-validation tests yielded by MLR model using *k*-fold CV. **B)** AUC values from 200 tests with resampled data and values yielded by 10-fold CV of resampled data. **C)** AUC from 200 tests of randomly split training and validation data. Top 5% and bottom 5% of data are shown in dots and horizontal bars of box, and whisker plots that indicate 5, 25, 50, 75, and 95% of the data.

**Table S1** Characteristics of tissue samples

| No. | Histological type | TNM | PD | Filling teeth | Missing teeth | Delayed teeth |
| --- | --- | --- | --- | --- | --- | --- |
| 1 | SCC | T2N1M0 | Y | 10 | 18 | 0 |
| 2 | SCC | T3N0M0 | Y | 13 | 3 | 3 |
| 3 | SCC | T3N0M0 | Y | 21 | 4 | 0 |
| 4 | SCC | T1N0M0 | N | 11 | 0 | 0 |
| 5 | SCC | T2N0M0 | Y | 11 | 5 | 0 |
| 6 | SCC | T4aN0M0 | Y | 2 | 26 | 0 |
| 7 | SCC | T2N1M0 | N | 0 | 28 | 0 |
| 8 | MM | T3N0M0 | - | - | - | - |
| 9 | SCC | T2N2bM0 | Y | 7 | 5 | 0 |
| 10 | MM | T3N0M0 | N | 0 | 28 | 0 |
| 11 | SCC | T1N0M0 | Y | 1 | 26 | 0 |
| 12 | SCC | T1N0M0 | Y | 16 | 0 | 1 |
| 13 | SCC | T4aN1M0 | Y | 2 | 2 | 0 |
| 14 | SCC | T1N0M0 | Y | 7 | 20 | 1 |
| 15 | SCC | T4aN0M0 | N | 0 | 0 | 0 |
| 16 | SCC | T2N0M0 | N | 5 | 0 | 0 |
| 17 | SCC | T2N0M0 | Y | 13 | 5 | 0 |
| 18 | SCC | T2N0M0 | Y | 8 | 20 | 0 |

MM: malignant melanoma; PD: periodontal disease; SCC: squamous cell carcinoma.

**Table S2** Metabolites of matched control and tumor tissue sets showing significantly different concentrations (adjusted *P* < 0.05).

| Metabolites | Control  (μmol/g) | | Tumor  (μmol/g) | | F.C. | *P*-value | Adjusted *P*-value | |
| --- | --- | --- | --- | --- | --- | --- | --- | --- |
|  | Ave. | S.D. | Ave. | S.D. |  |  |  |  |
| 1-Methylnicotinamide | 0.00461 | 0.00195 | 0.0259 | 0.0159 | 5.61 | 0.0000010 | 0.000013 | *** |
| Betaine | 0.0588 | 0.0187 | 0.148 | 0.174 | 2.52 | 0.0000010 | 0.000013 | *** |
| Asn | 0.118 | 0.0253 | 0.330 | 0.178 | 2.80 | 0.0000010 | 0.000013 | *** |
| Choline | 0.165 | 0.0663 | 0.415 | 0.155 | 2.52 | 0.0000010 | 0.000013 | *** |
| Asp | 0.248 | 0.129 | 1.38 | 0.579 | 5.58 | 0.0000010 | 0.000013 | *** |
| Pro | 0.273 | 0.0916 | 0.804 | 0.310 | 2.94 | 0.0000010 | 0.000013 | *** |
| Thr | 0.298 | 0.0979 | 0.702 | 0.232 | 2.36 | 0.0000010 | 0.000013 | *** |
| Glu | 1.22 | 0.512 | 5.15 | 1.86 | 4.24 | 0.0000010 | 0.000013 | *** |
| Ethanolamine phosphate | 1.99 | 4.331 | 4.89 | 6.99 | 2.46 | 0.0000010 | 0.000013 | *** |
| UDP-*N*-acetylglucosamine | 0.0749 | 0.236 | 0.518 | 0.764 | 6.92 | 0.0000014 | 0.000017 | *** |
| Hydroxyproline | 0.0202 | 0.0098 | 0.0536 | 0.0247 | 2.66 | 0.0000024 | 0.000018 | *** |
| Ile | 0.107 | 0.0337 | 0.296 | 0.122 | 2.78 | 0.0000024 | 0.000018 | *** |
| Tyr | 0.142 | 0.0483 | 0.320 | 0.144 | 2.25 | 0.0000024 | 0.000018 | *** |
| Val | 0.328 | 0.109 | 0.780 | 0.222 | 2.38 | 0.0000024 | 0.000018 | *** |
| Hypoxanthine | 0.379 | 0.148 | 0.816 | 0.268 | 2.15 | 0.0000024 | 0.000018 | *** |
| Gly | 1.01 | 0.547 | 2.97 | 1.20 | 2.93 | 0.0000024 | 0.000018 | *** |
| 2AB | 0.0414 | 0.0165 | 0.0936 | 0.0350 | 2.26 | 0.0000033 | 0.000024 | *** |
| Putrescine | 0.0119 | 0.00711 | 0.0924 | 0.0689 | 7.76 | 0.0000048 | 0.000025 | *** |
| Nicotinamide | 0.0408 | 0.0209 | 0.103 | 0.0462 | 2.53 | 0.0000048 | 0.000025 | *** |
| Phe | 0.139 | 0.0491 | 0.311 | 0.131 | 2.24 | 0.0000048 | 0.000025 | *** |
| Arg | 0.212 | 0.0681 | 0.424 | 0.153 | 2.00 | 0.0000048 | 0.000025 | *** |
| Leu | 0.263 | 0.0960 | 0.663 | 0.316 | 2.52 | 0.0000048 | 0.000025 | *** |
| Ser | 0.438 | 0.166 | 0.821 | 0.336 | 1.87 | 0.0000048 | 0.000025 | *** |
| SAM | 0.00404 | 0.00400 | 0.0198 | 0.0100 | 4.90 | 0.0000067 | 0.000031 | *** |
| His | 0.197 | 0.0681 | 0.319 | 0.0935 | 1.61 | 0.0000067 | 0.000031 | *** |
| Ala | 1.60 | 0.722 | 2.70 | 0.738 | 1.69 | 0.0000067 | 0.000031 | *** |
| Guanine | 0.0290 | 0.0243 | 0.0813 | 0.0346 | 2.81 | 0.000016 | 0.000070 | *** |
| Lys | 0.478 | 0.161 | 0.853 | 0.330 | 1.78 | 0.000021 | 0.000088 | *** |
| Glutathione (oxidized form) | 0.146 | 0.0852 | 0.353 | 0.181 | 2.42 | 0.000033 | 0.00013 | ** |
| Glutathione (reduced form) | 0.277 | 0.312 | 1.33 | 1.24 | 4.81 | 0.000033 | 0.00013 | ** |
| Fumarate | 0.176 | 0.238 | 0.294 | 0.351 | 1.67 | 0.000042 | 0.00016 | ** |
| Met | 0.0716 | 0.0352 | 0.156 | 0.0837 | 2.18 | 0.000065 | 0.00024 | ** |
| Kynurenine | 0.000768 | 0.00151 | 0.0293 | 0.0237 | 38.1 | 0.000074 | 0.00027 | ** |
| 3-Methylhistidine | 0.00853 | 0.00521 | 0.0187 | 0.0129 | 2.19 | 0.000081 | 0.00028 | ** |
| Phosphorylcholine | 0.123 | 0.0992 | 0.455 | 0.972 | 3.70 | 0.000081 | 0.00028 | ** |
| SAH | 0.00462 | 0.00312 | 0.0121 | 0.00605 | 2.62 | 0.000096 | 0.00032 | ** |
| GABA | 0.0097 | 0.00423 | 0.0335 | 0.0242 | 3.46 | 0.000132 | 0.00043 | ** |
| *N*-Acetylglucosamine 6-phosphate | 0.0195 | 0.0561 | 0.132 | 0.228 | 6.78 | 0.000146 | 0.00046 | ** |
| *N*-Acetylglucosamine 1-phosphate | 0.0167 | 0.0487 | 0.101 | 0.205 | 6.01 | 0.000177 | 0.00054 | ** |
| Gly-Gly | 0.00176 | 0.00212 | 0.00922 | 0.00610 | 5.24 | 0.000214 | 0.00063 | ** |
| GMP | 0.0423 | 0.121 | 0.227 | 0.373 | 5.37 | 0.000213 | 0.00063 | ** |
| 6-Phosphogluconate | 0.0232 | 0.0402 | 0.0985 | 0.153 | 4.25 | 0.000236 | 0.00067 | ** |
| Malate | 0.724 | 1.022 | 1.21 | 1.71 | 1.67 | 0.000256 | 0.00071 | ** |
| Trimethylamine *N*-oxide | 0.00783 | 0.00575 | 0.0125 | 0.00840 | 1.59 | 0.000271 | 0.00074 | ** |
| *N*-Acetylaspartate | 0.0463 | 0.0970 | 0.0929 | 0.122 | 2.01 | 0.000305 | 0.00078 | ** |
| UMP | 0.0905 | 0.245 | 0.266 | 0.390 | 2.93 | 0.000305 | 0.00078 | ** |
| AMP | 0.219 | 0.591 | 0.778 | 1.12 | 3.55 | 0.000305 | 0.00078 | ** |
| Citrulline | 0.0456 | 0.0208 | 0.106 | 0.0652 | 2.32 | 0.000362 | 0.00089 | ** |
| Lactate | 25.4 | 40.499 | 41.6 | 61.47 | 1.64 | 0.000362 | 0.00089 | ** |
| 3-Hydroxy-3-methylglutarate | 0.000739 | 0.00167 | 0.00816 | 0.0159 | 11.04 | 0.000419 | 0.0010 | ** |
| S7P | 0.0215 | 0.0348 | 0.0464 | 0.0694 | 2.16 | 0.000447 | 0.0011 | ** |
| Guanosine | 0.0410 | 0.0199 | 0.0678 | 0.0311 | 1.65 | 0.000504 | 0.0012 | ** |
| Argininosuccinate | 0.00288 | 0.00390 | 0.0232 | 0.0292 | 8.07 | 0.000892 | 0.0020 | ** |
| F1,6P | 0.0762 | 0.208 | 0.125 | 0.268 | 1.64 | 0.0010 | 0.0021 | ** |
| 3PG | 0.275 | 0.352 | 0.208 | 0.596 | 0.76 | 0.0013 | 0.0028 | ** |
| UDP-glucuronate | 0.0203 | 0.0703 | 0.0615 | 0.104 | 3.03 | 0.0014 | 0.0030 | ** |
| Spermidine | 0.00868 | 0.00608 | 0.0246 | 0.0256 | 2.83 | 0.0017 | 0.0035 | ** |
| Succinate | 0.356 | 0.721 | 0.628 | 1.05 | 1.76 | 0.0017 | 0.0035 | ** |
| Carnosine | 0.451 | 0.598 | 0.0573 | 0.131 | 0.13 | 0.0018 | 0.0036 | ** |
| 2-Hydroxyglutarate | 0.0108 | 0.0294 | 0.0291 | 0.0535 | 2.70 | 0.0018 | 0.0036 | ** |
| Taurine | 4.11 | 2.011 | 5.70 | 2.03 | 1.39 | 0.0022 | 0.0043 | ** |
| Cadaverine | 0.00181 | 0.00292 | 0.0137 | 0.0185 | 7.56 | 0.0024 | 0.0046 | ** |
| PEP | 0.0538 | 0.0685 | 0.0288 | 0.110 | 0.53 | 0.0029 | 0.0054 | ** |
| 5-Oxoproline | 0.214 | 0.314 | 0.401 | 0.681 | 1.88 | 0.0032 | 0.0061 | ** |
| *N*-Acetylputrescine | 0.00213 | 0.00343 | 0.00885 | 0.0159 | 4.15 | 0.0033 | 0.0062 | ** |
| Cysteine-glutathione disulphide | 0.0158 | 0.0150 | 0.0845 | 0.131 | 5.35 | 0.0038 | 0.0069 | ** |
| 1-Methylhistamine | 0.00423 | 0.00329 | 0.00848 | 0.00616 | 2.01 | 0.0039 | 0.0070 | ** |
| CMP | 0.0108 | 0.0336 | 0.0433 | 0.0779 | 4.00 | 0.0048 | 0.0085 | ** |
| UDP-glucose | 0.0427 | 0.134 | 0.118 | 0.229 | 2.77 | 0.0049 | 0.0085 | ** |
| *N*_1_-Acetylspermidine | 0.00353 | 0.00591 | 0.0229 | 0.0312 | 6.50 | 0.0054 | 0.0090 | ** |
| UDP | 0.0239 | 0.0799 | 0.0683 | 0.149 | 2.86 | 0.0054 | 0.0090 | ** |
| α-Aminoadipate | 0.0360 | 0.0200 | 0.0849 | 0.0845 | 2.36 | 0.0053 | 0.00902 | ** |
| *N*-Acetylneuraminate | 0.00587 | 0.0102 | 0.0199 | 0.0254 | 3.40 | 0.0057 | 0.0093 | ** |
| *cis*-Aconitate | 0.00848 | 0.0118 | 0.0185 | 0.0216 | 2.18 | 0.0074 | 0.012 | * |
| ADP | 0.221 | 0.506 | 0.423 | 0.636 | 1.92 | 0.0074 | 0.012 | * |
| 5-Aminovalerate | 0.0273 | 0.0327 | 0.0729 | 0.0776 | 2.67 | 0.0083 | 0.013 | * |
| Hypotaurine | 0.0623 | 0.0533 | 0.118 | 0.0847 | 1.89 | 0.0085 | 0.013 | * |
| Citrate | 0.293 | 0.449 | 0.454 | 0.486 | 1.55 | 0.0093 | 0.014 | * |
| Sarcosine | 0.00600 | 0.00878 | 0.0133 | 0.0105 | 2.22 | 0.014 | 0.021 | * |
| Gln | 2.33 | 1.421 | 3.23 | 1.42 | 1.38 | 0.019 | 0.028 | * |
| Trp | 0.0361 | 0.0096 | 0.0547 | 0.0370 | 1.52 | 0.021 | 0.031 | * |
| Histamine | 0.0877 | 0.0576 | 0.142 | 0.0955 | 1.62 | 0.021 | 0.031 | * |
| Pipecolate | 0.00175 | 0.00164 | 0.00290 | 0.00238 | 1.66 | 0.026 | 0.038 | * |
| Ornithine | 0.0539 | 0.0190 | 0.0779 | 0.0403 | 1.45 | 0.033 | 0.046 | * |
| DHAP | 0.0566 | 0.117 | 0.0268 | 0.0874 | 0.47 | 0.033 | 0.046 | * |

**Table S3** Metabolites of saliva samples from controls and oral cancers showing significantly different concentrations (adjusted *P* <0.05).

| Metabolites | Control (μM) | | Tumor (μM) | | F.C. | *P*-value | Adjusted *P*-value | |
| --- | --- | --- | --- | --- | --- | --- | --- | --- |
|  | Ave. | S.D. | Ave. | S.D. |  |  |  |  |
| Gly-Leu | 0.383 | 0.9561 | 1.80 | 2.27 | 4.70 | 0.0000023 | 0.00026 | ** |
| *N,N*-Dimethylglycine | 0.132 | 0.4283 | 0.483 | 0.466 | 3.67 | 0.000010 | 0.00036 | ** |
| Hexanoate | 12.8 | 12.745 | 67.5 | 98.1 | 5.28 | 0.0000086 | 0.00036 | ** |
| Octanoate | 0.765 | 0.5507 | 0.138 | 0.318 | 0.18 | 0.000014 | 0.00038 | ** |
| 4-Methylbenzoate | 6.22 | 12.871 | 36.9 | 42.6 | 5.93 | 0.000028 | 0.00062 | ** |
| 3PG | 2.29 | 4.1776 | 6.49 | 5.37 | 2.83 | 0.000024 | 0.00065 | ** |
| 3-Phenylpropionate | 7.35 | 9.3293 | 49.7 | 66.8 | 6.76 | 0.000053 | 0.00098 | ** |
| Isopropanolamine | 0.464 | 0.7196 | 1.56 | 1.57 | 3.36 | 0.000094 | 0.0015 | ** |
| SAM | 0.063 | 0.1275 | 0.315 | 0.400 | 4.99 | 0.00017 | 0.0023 | ** |
| 3-Phenyllactate | 1.92 | 2.9606 | 4.66 | 5.81 | 2.42 | 0.00022 | 0.0027 | ** |
| Urea | 1634 | 1408.8 | 556 | 932 | 0.34 | 0.00029 | 0.0032 | ** |
| Pipecolate | 0.565 | 0.7935 | 1.52 | 1.55 | 2.69 | 0.00046 | 0.0043 | ** |
| 3-(4-Hydroxyphenyl)propionate | 6.17 | 5.4571 | 27.8 | 32.4 | 4.51 | 0.00047 | 0.0043 | ** |
| Spermidine | 3.43 | 3.3281 | 7.36 | 5.37 | 2.15 | 0.00051 | 0.0043 | ** |
| Butanoate | 101 | 76.685 | 341 | 498 | 3.38 | 0.00063 | 0.0050 | ** |
| Met | 2.10 | 3.4549 | 6.64 | 9.91 | 3.17 | 0.00075 | 0.0056 | ** |
| 2-Hydroxy-4-methylpentanoate | 3.02 | 4.9555 | 7.80 | 10.6 | 2.58 | 0.00095 | 0.0062 | ** |
| 2-Hydroxypentanoate | 8.32 | 15.584 | 16.4 | 19.4 | 1.97 | 0.00093 | 0.0062 | ** |
| *N*-Acetylornithine | 0.144 | 0.3229 | 0.492 | 0.623 | 3.43 | 0.0012 | 0.0072 | ** |
| 2AB | 1.92 | 1.9948 | 6.77 | 12.4 | 3.52 | 0.0015 | 0.0089 | ** |
| *N*_8_-Acetylspermidine | 0.079 | 0.114 | 0.142 | 0.100 | 1.80 | 0.0022 | 0.012 | * |
| Guanosine | 0.516 | 1.9904 | 0.910 | 1.81 | 1.76 | 0.0023 | 0.012 | * |
| Val | 19.8 | 23.197 | 47.0 | 62.3 | 2.38 | 0.0023 | 0.012 | * |
| Trimethylamine *N*-oxide | 0.300 | 0.6855 | 0.761 | 1.18 | 2.54 | 0.0026 | 0.012 | * |
| Trp | 2.31 | 2.6839 | 5.18 | 5.90 | 2.24 | 0.0027 | 0.013 | * |
| 7-Methylguanine | 0.115 | 0.167 | 0.375 | 0.662 | 3.27 | 0.0034 | 0.014 | * |
| Gly-Gly | 1.04 | 2.0488 | 2.23 | 3.05 | 2.16 | 0.0033 | 0.014 | * |
| *γ*-Butyrobetaine | 3.55 | 3.1066 | 7.40 | 6.18 | 2.08 | 0.0032 | 0.014 | * |
| Ala-Ala | 1.44 | 1.4032 | 2.86 | 2.80 | 1.98 | 0.0041 | 0.016 | * |
| Hypoxanthine | 9.86 | 11.977 | 19.6 | 20.9 | 1.99 | 0.0053 | 0.020 | * |
| Ru5P | 4.94 | 3.7506 | 7.38 | 3.98 | 1.49 | 0.0058 | 0.021 | * |
| 1,3-Diaminopropane | 1.55 | 3.1614 | 2.86 | 4.10 | 1.85 | 0.0068 | 0.024 | * |
| Guanine | 1.26 | 1.0648 | 2.27 | 2.24 | 1.80 | 0.0090 | 0.030 | * |
| *β*-Ala | 2.73 | 2.0932 | 4.90 | 3.72 | 1.79 | 0.0090 | 0.030 | * |
| Taurine | 152 | 141.68 | 252 | 186 | 1.65 | 0.0094 | 0.031 | * |
| Choline | 15.7 | 14.09 | 24.1 | 17.5 | 1.54 | 0.0098 | 0.031 | * |
| 3-Hydroxybutyrate | 7.30 | 6.3135 | 11.3 | 6.90 | 1.54 | 0.011 | 0.035 | * |
| Cadaverine | 24.7 | 30.936 | 75.8 | 109 | 3.07 | 0.012 | 0.035 | * |
| O-Phosphoserine | 0.668 | 0.9129 | 2.06 | 2.48 | 3.08 | 0.012 | 0.036 | * |
| F6P | 2.54 | 7.0591 | 1.46 | 4.43 | 0.57 | 0.013 | 0.036 | * |
| *cis*-Aconitate | 0.374 | 0.6951 | 0.198 | 0.487 | 0.53 | 0.016 | 0.044 | * |
| *N*-epsilon-Acetyllysine | 0.232 | 0.4432 | 0.507 | 0.605 | 2.19 | 0.016 | 0.044 | * |
| Thr | 10.9 | 12.443 | 19.8 | 18.3 | 1.82 | 0.018 | 0.047 | * |

**Table S4** Salivary metabolites that show consistent changes in concentration over disease stages in saliva and tumor samples

| Metabolites | Oral cancer vs controls | | | | Stage I+II vs III+IV |
| --- | --- | --- | --- | --- | --- |
|  | AUC | 95% CI | | *P*-value | *P*-value |
| 3PG | 0.767 | 0.635 | 0.899 | 0.00030 | 0.48 |
| Pipecolate | 0.755 | 0.637 | 0.873 | 0.00056 | 0.98 |
| Spermidine | 0.751 | 0.626 | 0.876 | 0.00068 | 0.73 |
| Met | 0.744 | 0.628 | 0.861 | 0.00094 | 0.09 |
| SAM | 0.743 | 0.613 | 0.874 | 0.00098 | 0.34 |
| 2AB | 0.734 | 0.610 | 0.859 | 0.0015 | 0.09 |
| Trp | 0.722 | 0.599 | 0.844 | 0.0027 | 0.07 |
| Val | 0.722 | 0.601 | 0.843 | 0.0027 | 0.23 |
| Hypoxanthine | 0.706 | 0.583 | 0.830 | 0.0052 | 0.25 |
| Gly-Gly | 0.702 | 0.573 | 0.830 | 0.0063 | 0.22 |
| Trimethylamine *N*-oxide | 0.701 | 0.569 | 0.832 | 0.0065 | 0.68 |
| Guanine | 0.693 | 0.558 | 0.828 | 0.0089 | 0.54 |
| Guanosine | 0.693 | 0.557 | 0.828 | 0.0090 | 0.59 |
| Taurine | 0.690 | 0.558 | 0.822 | 0.0099 | 0.42 |
| Choline | 0.689 | 0.562 | 0.817 | 0.010 | 0.42 |
| Cadaverine | 0.685 | 0.554 | 0.816 | 0.012 | 1.0 |
| Thr | 0.674 | 0.540 | 0.808 | 0.018 | 0.15 |

**Table S5.** Parameters of the MLR model

| Parameter | Odds ratio | 95% CI | | Coefficient | 95% CI | | *P*-Value |
| --- | --- | --- | --- | --- | --- | --- | --- |
| SAM | 109.4 | 5.262 | 4011 | 4.695 | 1.661 | 8.297 | 0.0052 |
| Pipecolate | 1.852 | 1.118 | 3.564 | 0.616 | 0.112 | 1.271 | 0.0362 |
| (Intercept) | - | - | - | −1.889 | −2.882 | −1.062 | <.0001 |
